# Secondary traumatic stress and death anxiety in healthcare professionals: Moderating role of social support

**DOI:** 10.12669/pjms.39.5.7254

**Published:** 2023

**Authors:** Shazia Qayyum, Ayesha Tahir, Faiz Younas

**Affiliations:** 1 Shazia Qayyum, PhD. Associate Professor, Institute of Applied Psychology, University of Punjab, Lahore - Pakistan; 2 Ayesha Tahir, M.Phil. Institute of Applied Psychology, University of Punjab, Lahore - Pakistan; 3 Faiz Younas, M.Phil. Lecturer, Institute of Applied Psychology, University of Punjab, Quaid-e-Azam Campus, 54590, Lahore - Pakistan

**Keywords:** Secondary traumatic stress, Social support, Death anxiety, Healthcare professionals, Health psychology

## Abstract

**Background & Objective:**

It’s hard to deny the buffering impact of social support as it provides much-needed assistance to counter untoward circumstances that individuals face in their daily life. By focusing on the moderating role of social support, the present investigation studied the relationship between secondary traumatic stress and death anxiety that healthcare professionals encounter in their regular work life.

**Method:**

Through a cross-sectional correlational design, 200 participants were included from various hospitals in Lahore (from June-August, 2022) by employing a non-probability purposive sampling technique. They provided basic sociodemographic information along with their responses on self-reported questionnaires for the current investigation.

**Results:**

Results were analyzed through SPSS 21 which indicated that secondary traumatic stress had a positive association with death anxiety, unlike social support which had a negative relationship with death anxiety. Findings also revealed social support as a significant moderator for secondary traumatic stress and death anxiety.

**Conclusions:**

It can be concluded that increased social support could benefit healthcare professionals as it weakened the association between secondary traumatic stress and death anxiety. Other than academia and research, these findings have implications across a variety of professional settings including physical and mental healthcare professionals who can benefit from these indigenous findings.

## INTRODUCTION

Stress and death are two experiential realities that every individual will eventually go through in their lives.^1^ While every person experiences the effects of stress regularly, it’s probable that some of us are more stressed than others. Moreover, owing to their specific professional responsibilities and workplace dynamics, some individuals are more prone to develop certain forms of stress like secondary traumatic stress (STS), which explains the stress response brought on in caregivers after being exposed to traumatic material from clients. It is a class of normal and inevitable behaviours and feelings brought on by learning about a traumatic event that a close friend (or client) went through as well as the anxiety brought on by trying to or wanting to help a person who is traumatized or in pain.^2^

Similarly, death as a concept has been imagined throughout history as a driving force behind a great deal of artistic expression and philosophical investigation. The fear brought on by knowing that one is going to die is regarded as death anxiety (DA).^3^

It’s an undeniable fact that everyone will eventually pass away but it’s still hard to let accept this fundamental reality. Questions like when one will die, what will happen after death, and how anyone will die; are all the daily concerns of someone who experience death anxiety. Research shows that these concerns can potentially interfere with one’s daily living.^4^ Moreover, several in-depth investigations indicated that social support (SS) enabled people to handle stress better. The foremost components of social support are known as primary groups (included family and friends) and secondary groups (typically included external goal-oriented motivations); both of whom equip participants with various coping strategies.^5^

The main aims of the present study were to determine the connections among STS, SS, and DA in healthcare professionals and to further examine if SS might act as a moderator between STS and DA. A quick review of available literature suggests a lack of indigenous evidence regarding the potential role of SS between STS and DA. The foremost and widely accepted theory in this area of investigation is the Stress and Coping Social Support Theory, which contends that by bringing changes in how people process and deal with stressful events, SS can shield individuals from the negative health impacts of stress (also known as stress buffering). Stressful events receive negative appraisals from the individuals experiencing them directly or indirectly and many reported coping with them poorly. At times the experiences turn so upsetting and unpleasant that people tend to develop DA. Studies found stress safeguarding benefits for perceived SS provide proof for the stress and coping social support theory.^6^

An investigation undertook the effects of DA on STS disorder and mental health in individuals who had witnessed life-threatening situations that another person had experienced and revealed a solid positive association between STS and fear of dying.^7^

Furthermore, Bibi and Khalid addressed the connection between perceived SS and DA in breast cancer survivors and found that SS had a negative relationship with DA. Moreover, the SS helped the individuals feel less anxious about dying and speeded up their recovery.^8^

Simialrly, another study explored the connection between doctors’ STS, burnout, and psychological resilienc during the COVID-19 epidemic and found that psychological resilience had a negative association with STS disorder and burnout whereas STS had a strong, favourable link with burnout.^9^

Similarly, a Pakistani study identified the potential risks of high-risk infections and mental health issues, for medical and healthcare personnel exposed to and in close contact with confirmed and suspected coronavirus cases. Findings have suggested that it affected the decisions making ability of medical professionals along with a long-term negative impact on their general well-being (including psychological problems like anxiety, fear, panic attacks, psychological distress, stigma and avoidance of contact, depressive tendencies, sleep disturbances, helplessness, interpersonal social isolation from family SS, and worry about contagion exposure to their friends and family).^10^

**Fig.1:**
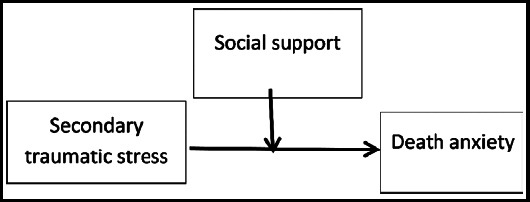
The hypothesized model presenting the linkage between secondary traumatic stress and death anxiety with the moderating role of social support.

Likewise, another study examined how people with chronic conditions such as cancer, hepatitis, cardiovascular disease, and diabetes perceived SS and worried about dying. Patients with chronic diseases might experience fewer thoughts of death with the use of psychosocial support and other services, which enabled them to function better and hasten their recovery.^1^ Based on the above-cited literature, we proposed the theoretical model for the present study as shown in Fig.1.

To investigate this model, we developed the following hypotheses:

- Secondary traumatic stress would have a significant negative relationship with social support and a positive relationship with death anxiety in healthcare professionals.
- Social support would have a negative relationship with death anxiety in healthcare professionals.
- Secondary traumatic stress would positively predict while social support would negatively predict death anxiety in healthcare professionals.
- Social support would moderate the relationship between secondary traumatic stress and death anxiety in healthcare professionals.

## METHODS

A cross-sectional correlational research design was used by employing a non-probability purposive sampling strategy that recruited 200 (suggested by G-power analysis) healthcare professionals from different hospitals of Lahore (during June-August 2022) with an age range of 25-35 years. Participants filled out a sociodemographic information sheet along with scales measuring the study variables. Since the data for this study was produced using a multi-scaled response format, reliability checks were carried out to make sure the primary constructs were consistent.^12^,^13^ Using reliability Cronbach alpha values, the internal consistency of each factor was evaluated that showed strong indices for The Secondary Traumatic Stress Scale (.97), the Death Anxiety Survey (.88) and The Multidimensional Scale of Perceived Social Support (.98). Data was analyzed through SPSS version 21.

### Ethical Approval:

This study was approved by Departmental Doctoral Program Committee (DDPC), Chairperson, Advanced Studies & Research Board (ASRB) & Vice-Chancellor, the University of Punjab (vide No. 4041/VC dated 26-05-2022) and mandated ethical guidelines for research were ensured..

## RESULTS

Table-I showed that secondary traumatic stress had a negative relationship with social support and a positive relationship with death anxiety. Also, social support was found to have a negative association with death anxiety. Table-I

**Table-I:** Correlations of Study Variables (N =200).

Variables	1	2	3
1. Secondary traumatic stress	-	-.98**	.86**
2. Social support		-	-.88**
3. Death anxiety			-

***Note:*** *p<.05

** p<.01.

The overall model explained 34% variance in Death Anxiety *F* (1, 193) =.26*, p<.001*. Table-II. The model-I explained 11% variance *F_change_*(1, 195) = 35.23, *p*<.001. whereas, model-II 16% variance *F_change_* (1, 194) = .10, *p*=.05. Model-III explained a 7% variance F*_change_* (1, 193) = 8.20, *p*<.001. Moreover, secondary traumatic stress and social support, both predicted death anxiety, while social support acted as a moderator between secondary traumatic stress and death anxiety.

**Table-II:** Social Support as a Moderator between Secondary Traumatic Stress and Death Anxiety (N = 200)

	Death Anxiety	

Variables	∆R^2^	β
Step-I	.11**	
***Secondary Traumatic Stress***		.17**
Step-II	.16*	
***Social Support***		-.19**
Step-III	.07***	
Secondary traumatic stress*social support		-.05***
Total *R^2^*	.34	

***Note:***

* p<.05;

** p<.01;

*** p<.001; β = Standardized Coefficient; ∆R2= R Square change; R2= R Square.

## DISCUSSION

This paper highlighted the relationship between secondary traumatic stress, social support and death anxiety in healthcare professionals. Findings indicated that STS and DA had a negative relationship with SS. Moreover, STS positively associated with DA, unlike SS which had a negative relationship with DA. Also, SS emerged as a significant moderator for STS and DA. This meant that if the healthcare professionals had more SS, then they would experience less STS and vice versa. These results were consistent with the previous studies. A study by Khawar, Aslam and Amir focused on the role of SS on individuals with chronic conditions also showed that those participants who reported fewer thoughts of death received psychosocial support and other services that enabled better functionality and speedy recovery.^11^ Similarly, Manning, Terte and Stephens also found that STS negatively predicted self-care and SS, while SS was also found to have a negative association with DA.^14^ Therefore, it can be inferred that receiving lesser SS from friends, family and co-workers could lead to greater DA. Furthermore, Uslu, Terzioglu and Koc studied the differences in the hopelessness and fear of dying experienced by hospitalized gynecologic cancer patients and the potential connections between those conditions and SS. Findings suggested that SS significantly reduced patients’ feelings of hopelessness and DA.^15^

Results of the current study indicated that STS had a significant positive association with DA was consistent with another study focused on identifying the potential risks involved in high-risk infections and mental health issues for healthcare personnels exposed to and in close contact with confirmed and suspected coronavirus cases. Findings indicated that the personnels reported experiencing physical and psychological strain since the arrival of COVID-19 in Pakistan, deteriorating their mental well-being.^10^ Likewise, another study by Hoelterhoff and Chung found that those who had witnessed a life-threatening event encountered by another individual showed a solid positive association between STS and fear of dying.^7^

A study looking at how certain work-related characteristics predicted STS and burnout, as well as how much SS helped to lessen both of these occupational stress syndromes found that to minimize any potentially harmful repercussions of their job, counsellors needed to be aware of both the inner dynamics of their clients.^16^

Secondly, the current study revealed that STS positively predicted DA whereas SS negatively predicted DA. As far as indigenious settings are concerned, these findings provided evidence to an unexplored research dynamics, however these dynmaics had been studied internationally and the current findings were consistent with the findings of Martz who concluded that STS predicted DA in workers dealing with individuals with disabilities.^17^ Similarly, findings further indicated that DA (after adjusting for demographic and disability-related factors) significantly predicted several STS reactions in people who cared for people with spinal cord injuries, revealing SS as a negative predictor of DA.^18^ Likewise, Naderi and Hajihasani looked at how elderly people’s perceptions of SS and spiritual health related to their DA and reported that their fear of dying was negatively predicted by their spiritual well-being and their perceived familial social support.^19^

Yet another study explored the factors influencing doctors’ fear of dying while they were on duty in coronavirus wards or quarantine facilities. Results showed that increased duty hours and a heavy workload led to more fatigue and anxiety about dying.^20^

Thirdly, present results showed that SS led to DA after it was found to have a negative association with STS. Once again, this analysis was done for exploration purposes as there was no any previous literature indicating the moderating role of SS between STS and DA. One of the reasons seems to be the fact that STS is an under-explored research construct, especially in the indigenous context. Although, few studies have investigaxted the moderating effect of SS on DA like Kagan who focused on the moderating effects of perceived SS in the relationship between nurses’ psychological discomfort and worry about dying. Findings suggested that nurses who experienced less SS had higher levels of psychological discomfort and lower levels of DA.^21^

This study highlighted the significance of social support for healthcare professionals that are usually taken for granted but has taken an emotional toll on their mental well-being. It also emphasized the moderating role of SS between STS and DA. This calls for take proactive initatives for the mental well-being of the healthcare professionals, as it also has a detrimental impact on their patients.

### Limitations & Suggestions:

This study has some of the limitation as the sample size was relatively small. It reduces the chances for the generalizability of the research. The sample was restricted to only government hospitals of Lahore. However, recruiting sample from healthcare professionals of other cities of Pakistan as well as from private sector would enhance its external validity. Moreover, awareness seminars and workshops can be organized for healthcare professionals that can equip participants with the coping strategies to deal with these challenging situations so that they can manage their professional resposnibilities without compromising their own mental health.

## CONCLUSIONS

Healthcare professionals play an undeniably significant role in keeping the well-being of the citizens. While they have to render selfless efforts to deal with a variety of patients in their professional lives, they also have to bear secondary trauma in cases of deteriorating health and eventual death regularly. This calls for steady and consistent SS from their families, friends and co-workers as they navigate through the experiences of STS and DA. Overall findings highlighted the importance of SS as it was found to moderate the association between STS and DA. Moreover, result indicated existence of negative correlation between STS and SS as well as between SS and DA. However, a positive association between STS and DA was also found. Lastly, STS positively predicted DA whereas SS negatively predicted DA.

### Authors’ contributions:

**SQ:** Conceptualization, method, data collection, analysis. She is also responsible and accountable for the accuracy and integrity of this work.

**AT:** Conceptualization, Literature review, method, data collection, analysis

**FY:** Literature review, critical analysis, article writing, article revisions

All authors read, revised and approved the final manuscript.
